# NMDA receptor antagonists reduce amyloid-*β* deposition by modulating calpain-1 signaling and autophagy, rescuing cognitive impairment in 5XFAD mice

**DOI:** 10.1007/s00018-022-04438-4

**Published:** 2022-07-09

**Authors:** Júlia Companys-Alemany, Andreea L. Turcu, Marion Schneider, Christa E. Müller, Santiago Vázquez, Christian Griñán-Ferré, Mercè Pallàs

**Affiliations:** 1 Pharmacology Section, Department of Pharmacology, Toxicology, and Therapeutic Chemistry. Faculty of Pharmacy and Food Sciences, Institut de Neurociències, Universitat de Barcelona (NeuroUB), Av. Joan XXIII 27-31, 08028 Barcelona, Spain; 2 Laboratory of Medicinal Chemistry (CSIC Associated Unit), Department of Pharmacology, Toxicology, and Therapeutic Chemistry. Faculty of Pharmacy and Food Sciences and Institute of Biomedicine (IBUB), University of Barcelona, Av. Joan XXIII, 27-31, 08028 Barcelona, Spain; 3 PharmaCenter Bonn, Pharmaceutical Institute, Pharmaceutical and Medicinal Chemistry, University of Bonn, 53121 Bonn, Germany

**Keywords:** Alzheimer’s disease, NMDA receptor, NMDAR antagonist, Neurodegeneration, Amyloid plaques, p-Tau, Autophagy, Apoptosis

## Abstract

Overstimulation of *N*-methyl-d-aspartate receptors (NMDARs) is the leading cause of brain excitotoxicity and often contributes to neurodegenerative diseases such as Alzheimer’s Disease (AD), the most common form of dementia. This study aimed to evaluate a new NMDA receptor antagonist (UB-ALT-EV) and memantine in 6-month-old female 5XFAD mice that were exposed orally to a chronic low-dose treatment. Behavioral and cognitive tests confirmed better cognitive performance in both treated groups. Calcium-dependent protein calpain-1 reduction was found after UB-ALT-EV treatment but not after memantine. Changes in spectrin breakdown products (SBDP) and the p25/p35 ratio confirmed diminished calpain-1 activity. Amyloid β (Aβ) production and deposition was evaluated in 5XFAD mice and demonstrated a robust effect of NMDAR antagonists on reducing Aβ deposition and the number and size of Thioflavin-S positive plaques. Furthermore, glycogen synthase kinase 3β (GSK3β) active form and phosphorylated tau (AT8) levels were diminished after UB-ALT-EV treatment, revealing tau pathology improvement. Because calpain-1 is involved in autophagy activation, autophagic proteins were studied. Strikingly, results showed changes in the protein levels of unc-51-like kinase (ULK-1), beclin-1, microtubule-associated protein 1A/1B-light chain 3(LC3B-II)/LC3B-I ratio, and lysosomal-associated membrane protein 1 (LAMP-1) after NMDAR antagonist treatments, suggesting an accumulation of autophagolysosomes in 5XFAD mice, reversed by UB-ALT-EV. Likewise, treatment with UB-ALT-EV recovered a WT mice profile in apoptosis markers Bcl-2, Bax, and caspase-3. In conclusion, our results revealed the potential neuroprotective effect of UB-ALT-EV by attenuating NMDA-mediated apoptosis and reducing Aβ deposition and deposition jointly with the autophagy rescue to finally reduce cognitive alterations in a mice model of familial AD.

**Supplementary Information:**

The online version contains supplementary material available at 10.1007/s00018-022-04438-4.

## Introduction

*N*-methyl-d-aspartate receptors (NMDARs) are ionotropic glutamate receptors that play an essential role in the central nervous system (CNS), fundamentally in synaptic transmission and plasticity [1]. Nevertheless, the overactivation of NMDARs is a crucial factor that promotes the increase of intracellular Ca^2+^ levels leading to synaptic dysfunction and neuronal loss [2]. Hence, NMDARs’ dysfunction was associated with neurodegenerative disorders such as Alzheimer’s Disease (AD), supporting the rationale of memantine in AD therapy [3, 4].

AD is still an insufficiently understood progressive and neurodegenerative age-related disease, and is the most common form of dementia, and is predicted to become a global epidemic by 2050 [5–7]. Neuropathologically, the extracellular accumulation of the amyloid-β (Aβ) protein and the intracellular accumulation of hyper-phosphorylated tau (p-tau) protein are defined as the main characteristic hallmarks of the disease followed by synaptic loss mainly in the hippocampus [8, 9]. In this regard, it is interesting to note that activation of glycogen synthase kinase 3β (GSK3β) has a role in the hyper-phosphorylation of tau at most of its sites, promoting neurofibrillary tangles (NTFs) and neuronal dysfunction [10].

Several lines of evidence showed that the overactivation of NMDARs in AD is responsible for the expression alteration of several proteins such as calpains, Ca^2+^/calmodulin-dependent protein kinase II (CaMKII), GSK3β, among others, all of them having important roles in apoptosis and neurodegeneration [11–14]. Therefore, the modulation of NMDARs led to changes in different apoptotic proteins such as calpain and caspase proteases [15].

Autophagy processes eliminate intracellular organelles and can remove damaged aggregated proteins such as Aβ. Defective autophagy has been implicated in AD pathogenesis [16]. Both Aβ and tau accumulations can be eliminated by autophagy, suggesting that potentiation of this lysosomal process could be a treatment for AD [17–19]. Furthermore, neuronal autophagy also participates in neurotransmitter release, presynaptic assembly, axonal growth, and dendritic spine density formation [16]. Thus, the optimization of autophagy could potentially improve synaptic signaling in AD.

Memantine is an uncompetitive low-affinity NMDAR antagonist and is one of the few symptomatic treatments approved by Drug Administrations (EMA/FDA) [20]. The reduction of NMDARs overactivation, and the blockade of extrasynaptic NMDARs in front of synaptic ones by memantine, demonstrated the capacity to rescue memory deficits as well as protect neurons from Aβ pathology [21, 22]. Besides, many other processes involved in AD have been described to be influenced by the action of memantine, such as tau hyper-phosphorylation or apoptosis [11–13]. This treatment strategy has shown much effectiveness in AD pathology in different animal models [3, 23–25], even though clinical trials do not show the same potential effects seen in preclinical studies [26, 27]. Nonetheless, a growing body of evidence suggests that targeting NMDARs could help prevent or slow AD progression. Consequently, in recent years, increasing attention has been paid to identifying new NMDAR antagonists to enhance the effects of memantine [28, 29]. Recently, we have designed and characterized by in vitro and in vivo experiments a novel polycyclic amine, UB-ALT-EV, a voltage-dependent, moderate-affinity (IC_50_: 1.9 μΜ), uncompetitive NMDAR antagonist [30].

**Fig. 1:**
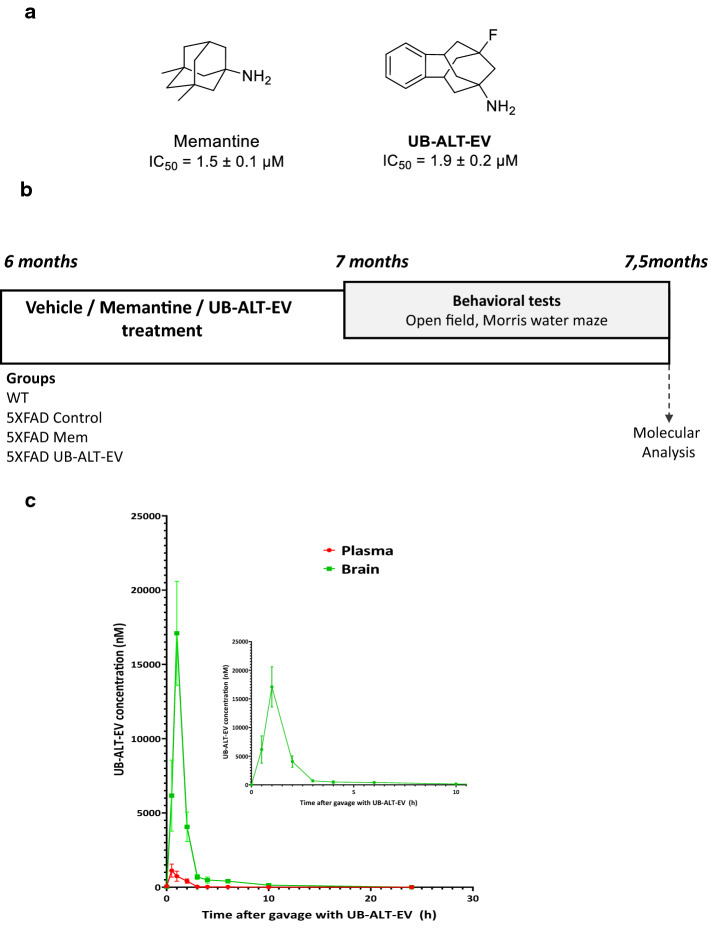
Chemical structures for UB-ALT-EV and memantine (**a**). Scheme of experimental design (**b**). Mean plasma and brain concentration − time profile of UB-ALT-EV in CD10 mice after per os administration. Inset depicts mean brain concentration − time profile of UB-ALT-EV in CD10 mice after per os administration. UB-ALT-EV (5 mg/kg; 10 mL/kg) was administered by oral gavage in mice. Data are shown as the mean ± SD (*n* = 3). The calculated parameters were (i) for plasma: *T*_max_ = 45 min, *C*_max_ = 1.12 ± 0.76 µM, elimination half-life = 1.5 h; (ii) brain: *T*_max_ = 56 min, *C*_max_ = 17.10 ± 6.06 µM, elimination half-life = 5 h

The 5XFAD strain is a well-established and suitable transgenic AD mouse model expressing five familial mutations of human AD. It also exhibits early onset cognitive impairment, starting at 3-month-old, including emotional disturbances. Moreover, Aβ plaque formation and gliosis starting at 2-month-old [31] have been described. Those events are accompanied by tau hyper-phosphorylation and synaptic dysfunction starting at 4- and 6-month-old, respectively [32]. Likewise, NMDARs’ dysfunction in the 5XFAD model has been described, correlating with cognitive impairment and Aβ accumulation [33, 34]. However, the effects of memantine or other NMDAR antagonists on neurodegenerative markers in 5XFAD mice are poorly described [23, 35]. It should be noted that the incidence of AD is higher in women than in men, so the study of a new treatment in female mouse models is mandatory to focus on the therapeutic usefulness in this sex [36]. Thus, the current work aimed to further evaluate the effects of chronic oral treatment of a new NMDAR antagonist, UB-ALT-EV (9-fluoro-5,6,8,9,10,11-hexahydro-*7H*-5,9:7,11-dimethanobenzo[9]annulen-7-amine hydrochloride), compared to memantine (Fig. 1a), in female 5XFAD mice. In addition, we focus on unveiling the molecular pathways modified by blocking NMDARs’ signaling beyond calcium entry blockade.

## Methods

### Animals and treatment

Female Wild-Type (WT) and 5XFAD mice (*n* = 43) at 6 months of age were used to perform the cognitive tests followed by molecular analysis. The animals were randomly divided into four groups: Wild-Type (WT) group (*n* = 14), 5XFAD Control group (Control) (*n* = 8), 5XFAD treated with memantine groups (Mem) (*n* = 11), and 5XFAD treated with UB-ALT-EV group (UB-ALT-EV) (*n* = 10). The sample size for the intervention was chosen following previous studies in our laboratory and using one of the available interactive tools (http://www.biomath.info/power/index.html) designed to estimate the required sample size to achieve adequate power. Animals had free access to food and water under standard temperature conditions (22 ± 2 °C) and 12 h:12 h light–dark cycles (300 lx/0 lx).

The experimental design is shown in Fig. 1b. Memantine and UB-ALT-EV were dissolved in aqueous 1.8% 2-hydroxypropyl-β-cyclodextrin and administered at 5 mg/kg/day through drinking water for 4 weeks and during the behavioral tests. The doses of the compounds were recalculated weekly by considering the daily water consumption in each cage and by monitoring the body weight of the animals weekly. The average daily water consumption per animal was 5 mL/day, and no differences were found between the groups. Also, the body weight of the animals was not significantly different during the whole treatment period (Fig. S1). The dosage of NMDAR antagonists was chosen based on published studies using AD mouse models [21, 28, 37]. Studies were performed by the Institutional Guidelines for the Care and Use of Laboratory Animals established by (European Communities Council Directive 2010/63/EU and Guidelines for the Care and Use of Mammals in Neuroscience and Behavioral Research, National Research Council 2003) and were approved by the Animal Experimentation Ethics Committee (CEEA) at the University of Barcelona. All efforts were made to reduce the number of animals and their suffering.

### Pharmacokinetic analysis

UB-ALT-EV was dissolved in 10% of 2-hydroxypropyl-*β*-cyclodextrin in physiological saline. Mice were monitored for signs of pain or distress between drug administration and euthanasia. The pharmacokinetic study was carried on in 27 male CD1 mice (Envigo Laboratories) with a body weight between 40 and 50 g (*n* = 3 per group), randomized to be included in the treated or control groups. Animals had free access to food and water under standard temperature conditions (22 ± 2 °C) and 12 h:12 h light–dark cycles (300 lx/0 lx). The drug formulation was prepared on the day of the study. The vehicle was 10% of 2-hydroxypropyl-*β*-cyclodextrin, (CAS 128446-35-5) Sigma-Aldrich (Ref.332607-25G). Mice were orally treated by gavage with a single dose of 5 mg/kg of the drug. The volume of administration was 10 mL/kg and the required volume was calculated before each administration according to animal weight. Mice were anesthetized and sacrificed by cervical dislocation, and blood samples (0.5–0.8 mL) were collected from animals at different time points (0 h, 0.5 h, 1 h, 2 h, 3 h, 4 h, 6 h, 10 h, and 24 h after drug administration) in tubes with serum gel and clotting activator (Sarstedt Micro tube 1.1 mL Z-Gel). Blood samples were centrifuged at 10,000 rpm for 5 min to obtain plasma that was stored at − 20 °C until analysis. Experimental procedures were in line with the Directive 2010/63/EU and approved by the Institutional Animal Care and Generalitat de Catalunya (#10291, 1/28/2018). Frozen plasma samples were defrosted, and 50 µL of cold acetonitrile containing 0.1% formic acid was added to 50 µl of plasma sample. After homogenization, followed by centrifugation (15 min at 15,000 rpm), the supernatant was transferred to an HPLC vial, and 4 µL were injected. Frozen brain samples were weighed after thawing. Then, 1 mL of acetonitrile containing 0.1% formic acid was added to each brain sample. The mixture was treated in a Tissuelyser (Qiagen, Germany) at 50 Hz for 5 min, followed by centrifugation for 15 min at 15,000 rpm. Then, 200 µL of each sample were transferred into HPLC vials, and 2.8 µL of the sample were injected. Calibration samples were run before, during and after the actual samples, injecting a volume of 2 µL. Mass spectra were recorded on a QTrap 6500 + (Sciex, Darmstadt, Germany) with an ESI-source coupled to an HPLC 1290 Infinity (Agilent, Waldbronn, Germany) using a Kinetex C18 PolarRP column (Phenomenex). The column temperature was 30 °C. An HPLC gradient was run starting with 98% water containing 0.1% formic acid and 2% acetonitrile containing 0.1% formic acid to 100% acetonitrile containing 0.1% formic acid within 2.2 min followed by flushing the column with acetonitrile containing 0.1% formic acid for 1.3 min and subsequent equilibration for 1.5 min. Sample solutions (2 µL each) were injected applying a flow rate of 0.6 mL/min. Dihydrocodeine tartrate was used as an internal standard (100 nM). Multiple reaction monitoring (MRM) was applied for quantification. Two different methods were employed: (i) MRM 232 $\to$ 155, (ii) MRM 232 $\to$ 215 (internal standard, MRM 302 $\to$ 199). Both methods yielded comparable results, and all determined data were included in the calculations.

### Cognitive tests

#### Open field test

In brief, the open field test (OFT) was performed using a wall-enclosed area as previously described [38]. The ground was divided into two defined as the center and peripheral areas. Behavior was evaluated with SMART^®^ ver.3.0 software, and each test was recorded for later evaluation using a camera located above the apparatus. Mice were located at the center and allowed to explore the white polywood box (50 × 50 × 25 cm) for 5 min. Then, the animals were returned to their home cages, and the OFT apparatus was cleaned with 70% ethanol (EtOH). The parameters measured included center time duration, rearings, defecations, and locomotor activity, calculated as the sum of global distance moved in the arena for 5 min.

#### Morris water maze

The Morris water maze (MWM) is a cognitive procedure extensively used to study spatial memory and learning [39]. An open circular pool (100 cm × 50 cm) of opaque water, which contains white latex paint (temperature maintained at 24 °C ± 1), and an escape platform submerged 1.5 cm below the water level (in the middle of one quadrant). The task was performed across seven consecutive days. On the first day, the platform was not present in the pool to allow the mice to swim and explore the pool for 60 s. The learning phase was conducted from the second day until day 6th, and each group was trained in 5 daily trials of 60 s. Each trial placed the animal in the water in five different starting points (set at NE, E, SE, S, and SW) and allowed the animal to swim until it found the platform. When the animal found the platform, the investigator left the animal to remain on the platform for 30 s to allow for spatial orientation. If the animal could not reach the platform in 60 s, the investigator guided the animal until the platform and left the animal for 30 s on it. One minute was allowed between trials, and every trial started from different locations in the pool. On the 7th day, the memory test was performed, the platform was removed from the pool and only one trial of 60 s was conducted. The animals swimming path patterns were recorded by a camera located above the pool, and data were analyzed with SMART v 3.0 software from Panlab. The parameters measured were the mean distance traveled to the platform during the learning phase, the mean distance traveled in the four quadrants, the mean distance traveled to the platform zone the trial day, the number of entries in the platform quadrant, and the number of entries in the platform zone.

### Brain tissue preparation

After 3 days of the cognitive and memory tests, all mice groups were euthanized by cervical dislocation and brains were immediately removed from the skull. For molecular experiments, the hippocampi were isolated and frozen in powdered dry ice. They were maintained at − 80 °C for further use for protein extraction and RNA isolation. For protein extraction, tissue samples were homogenized in lysis buffer (50-mM Tris–HCl pH 7.4, 150-mM NaCl, 5-mM EDTA, and 1% Triton X-100) containing phosphatase and protease inhibitors cocktails (Cocktail II, Sigma-Aldrich, St. Louis, MO, USA). Total protein levels were obtained, and protein concentration was determined by the method of Bradford. For thioflavin-S staining, mice were anesthetized (ketamine 100 mg/kg and xylazine 10 mg/kg, intraperitoneally) and then perfused with 4% paraformaldehyde (PFA) diluted in 0.1 M phosphate buffer solution intracardially. Brains were removed and postfixed in 4% PFA overnight at 4 °C. Afterward, brains were changed to PFA + 15% sucrose. Finally, the brains were frozen on powdered dry ice and stored at -80 °C until sectioning. Brain coronal sections of 30 μm were obtained (Leica Microsystems CM 3050S cryostat, Wetzlar, Germany) and kept in a cryoprotectant solution at − 20 °C until use.

### Protein level determination by western blotting

For Western Blotting (WB), aliquots of 15 μg of hippocampal protein extraction per sample were used. Protein samples were separated by sodium dodecyl sulfate–polyacrylamide gel electrophoresis (SDS-PAGE) (8–20%) and transferred onto polyvinylidene difluoride (PVDF) membranes (Millipore). Afterward, membranes were blocked in 5% non-fat milk in Tris-buffered saline (TBS) solution containing 0.1% Tween 20 TBS (TBS-T) for 1 h at room temperature (RT), followed by overnight incubation at a 4 °C with the primary antibodies listed in Table S1. Then, membranes were washed and incubated with secondary antibodies for 1 h at RT. Immunoreactive proteins were viewed with the chemiluminescence-based detection kit, following the manufacturer’s protocol (ECL Kit, Millipore), and digital images were acquired using ChemiDoc XRS + System (Bio-Rad). Semi-quantitative analyses were done using ImageLab software (Bio-Rad), and results were expressed in Arbitrary Units (AU), considering control protein levels as 100%. Protein loading was routinely monitored by immunodetection of glyceraldehyde-3-phosphate dehydrogenase (GAPDH).

### Thioflavin-S staining

Thioflavin-S staining was performed using three animals per group. Three brain sections per animal were first rehydrated at room temperature by 5 min incubation in PBS. Next, brain sections were washed 1 min each in 50%, 70%, and 80% ethanol and incubated with 0.3% Thioflavin-S (Sigma-Aldrich) solution for 10 min at RT in the dark. Subsequently, these samples were submitted to washes in 1 min series of 80%, 70%, and 50% and finally, one last wash of 5 min in PBS. Then, slides were mounted with Fluoromount-GTM (EMS, Hatfield, NJ, USA) and allowed to dry overnight. Image acquisition was performed with a fluorescence laser microscope (Olympus BX51, Germany) using 4X and 20X objectives, and images were analyzed using ImageJ software. Similar and comparable histological areas were selected for plaque quantification, focusing on the adjacent positioning of the whole cortical area and the hippocampus. Each image was converted to 8-bit greyscale, thresholded to a linear scale, and the number of particles and the percentage of area covered by thioflavin-S were calculated and then averaged from three different sections from each animal.

### Amyloid-β protein level quantification by ELISA

Aβ_40_ and Aβ_42_ protein levels were analyzed by ELISA with the human amyloid-β_40_ ELISA Kit (Invitrogen, #KHB3481; Thermo Fisher) and the human amyloid-β_42_ ultrasensitive ELISA Kit (Invitrogen, #KHB3441), respectively. All procedures followed the manufacturer’s instructions.

### Data acquisition and statistical analysis

Data analysis was conducted using GraphPad Prism ver. 8 statistical software. Data are expressed as the mean ± standard error of the mean (SEM) of at least three samples per group. Data have been analyzed using the Shapiro–Wilk test for normality to ensure that parametric tests can be used. One-way analysis of variance (ANOVA) was followed by Tukey post hoc analysis or two-tailed Student’s *t* test when necessary. In case of MWM learning curve, repeated-measures ANOVA was performed. Statistical significance was considered when p values were < 0.05. The statistical outliers were determined with Grubbs’ test and, when necessary, were removed from the analysis. The cognitive analysis was performed blindly. An experimenter unaware of the treatment groups performed the tests and recorded the number of animals. Another experimenter analyzed the videos and the behavioral scoring.

## Results

### Pharmacokinetic properties of UB-ALT-EV in mice

To characterize the pharmacokinetic profile of UB-ALT-EV after oral administration, its bioavailability (plasma and brain levels) was assessed in mice. Following a single oral administration of UB-ALT-EV (5 mg/Kg), absorption of UB-ALT-EV from the gastrointestinal tract was fast, reaching a maximum plasma concentration (*C*_max_) of 1.12 ± 0.76 µM after 45 min. Interestingly, a much higher concentration of UB-ALT-EV was found in the brain (Fig. 1c inset) compared to plasma, indicating a fast and very high blood–brain barrier (BBB) penetration. A *C*_max_ of 17.10 ± 6.06 µM was determined in the brain, which was reached after about 1 h (*T*_max_ = 56 min). Thus, the *C*_max_ in the brain was 15-fold higher than that in plasma. A brain/plasma ratio of 32 was observed after 1 h at *C*_max_ (brain) (Fig. 1c).

### Improvement in locomotion, anxiety-like behavior, and spatial memory after treatment with UB-ALT-EV

**Fig. 2:**
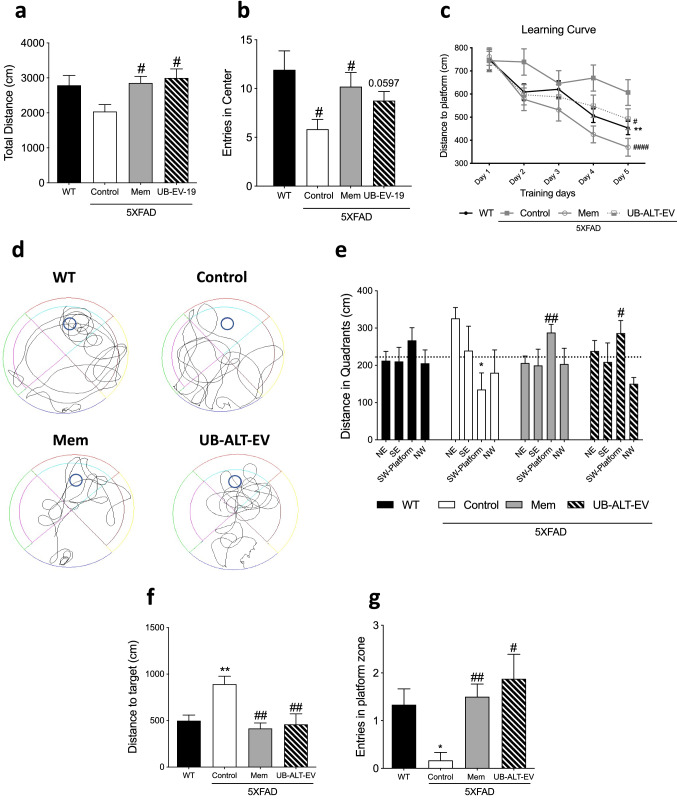
Results of OF: total distance (**a**), and entries in center zone (**b**). For Morris water maze: learning curve (**c**), representative swim paths patterns for each group (**d**), distance traveled in quadrants (**e**), distance to target of the test day (**f**)**,** and entries in platform zone (**g**). Values are the mean ± standard error of the mean (SEM); (*n* = 14 for WT, n = 8 for Control, *n* = 11 for Mem and n = 10 for UB-ALT-EV). For WT *vs*. 5XFAD control groups, data were analyzed using a two-tailed Student’s t test, and for 5XFAD groups, a standard one-way ANOVA followed by Tukey post hoc analysis was performed. In the case of MWM learning curve, a repeated-measures ANOVA was performed. *p < 0.05; **p < 0.01 for WT vs. Control. ^#^*p* < 0.05; ^##^*p* < 0.01; for Mem or UB-ALT-EV vs. Control. For the distance in quadrants case: *p < 0.05 for Quadrant SW WT vs. Quadrant SW Control. ^#^*p* < 0.05; ^##^*p* < 0.01; for Quadrant SW Mem or UB-ALT-EV vs. Quadrant SW Control

OFT evaluation revealed an improvement of locomotor activity in treated groups compared to the 5XFAD Control group via the analysis of the total distance (Fig. 2a). The analysis of the entries in the OFT center zone, as evaluation of anxiety-like behavior, showed significantly fewer entries for 5XFAD Control group in comparison with WT group (Fig. 2b). Interestingly, both NMDAR antagonist-treated groups increased center entries (Fig. 2b).

The MWM is used to assess spatial memory. After the training period, the learning curve (Fig. 2c) revealed that all groups learned the platform’s location except the 5XFAD control group, suggesting a learning deficits recovery after NMDAR antagonist treatment. On the trial day, the swimming paths were recorded, and each group’s representative swimming path pattern is shown (Fig. 2d). This figure revealed that WT group, along with memantine and UB-ALT-EV-treated groups, spent most of the time trying to find the platform in the correct quadrant, while the 5XFAD control group showed an erratic swim-pattern. In this line, all the groups except the 5XFAD Control group traveled more distance in the platform quadrant than in any other quadrant (Fig. 2e). When the distance to reach the platform was evaluated, only 5XFAD control group did travel significantly more distance when compared to other groups (Fig. 2f). Moreover, WT group, memantine, and UB-ALT-EV groups showed significantly more entries into the platform zone than the 5XFAD control group (Fig. 2g). These results suggest that memantine and UB-ALT-EV protected against impairments in spatial memory caused by the 5XFAD mutations.

### Chronic administration UB-ALT-EV prevented calcium-dependent proteins’ activation

**Fig. 3:**
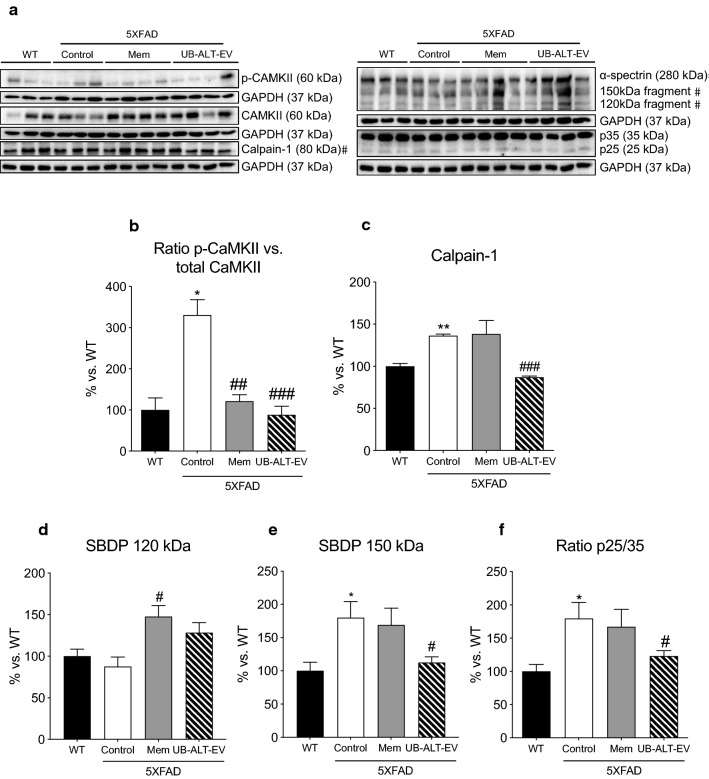
Representative western blot (**a**) and quantifications for ratio p-CAMKII vs. total CAMKII (**b**)**,** Calpain-1 (**c**)**,** SBDP 120 kDa (**d**)**,** SBDP 150 kDa (**e**)**,** and ratio p25/35 (**f**). Values in bar graphs are adjusted to 100% for protein levels of the wild type (WT). Values are the mean ± Standard error of the mean (SEM) (*n* = 3 for WT and Control groups and *n* = 4 for Mem and UB-ALT-EV groups). For WT vs. 5XFAD control groups, data were analyzed using a two-tailed Student’s t test, and for 5XFAD groups, a standard one-way ANOVA followed by Tukey post hoc analysis was performed. *p < 0.05; **p < 0.01; for WT vs. Control. ^#^*p* < 0.05; ^##^*p* < 0.01; ^###^*p* < 0.001 for Mem or UB-ALT-EV vs. Control

Considering the relationship between intracellular calcium levels and NMDAR dysfunction, calcium-dependent proteins CaMKII and calpain-1 were evaluated. Regarding the CaMKII activation, we found significantly increased p-CaMKII levels in 5XFAD; both memantine and UB-ALT-EV treatments reduced p-CaMKII levels (Fig. 3a, b). Likewise, compared to WT mice, the 5XFAD control group showed significant higher calpain-1 protein levels that were significantly reduced after UB-ALT-EV treatment, but not in the case of memantine-treated group (Fig. 3a, c).

Pursuing the UB-ALT-EV effects on calpain-1 protein levels, we evaluated α-spectrin breakdown products (SBDP), a target of this protease. Accordingly, results revealed that 5XFAD control mice presented significant higher protein levels of SBDP 150 kDa fragment, a marker of calpain activation, when compared to WT mice group, being diminished by UB-ALT-EV treatment (Fig. 3a, e). In addition, when the calpain target p35 was evaluated through the p25/35 ratio, 5XFAD mice exhibited significantly higher levels when compared to WT (Fig. 3a, f), further indicating a calpain-1 activation. Interestingly, UB-ALT-EV, but not memantine, reduced SBPD 150 kDa and p25/p35 ratio compared to 5XFAD control mice (Fig. 3a, d–f). All these findings agree with the hypothesis by reducing NMDAR overactivation, and all the calcium-dependent proteins would reduce their overactivity, then contributing to stopping the neurodegenerative process.

### Reduced APP protein processing and tau kinase activation promotes a diminution in tau pathology and increases synapsin I level in 5XFAD mice after UB-ALT-EV treatment

**Fig. 4:**
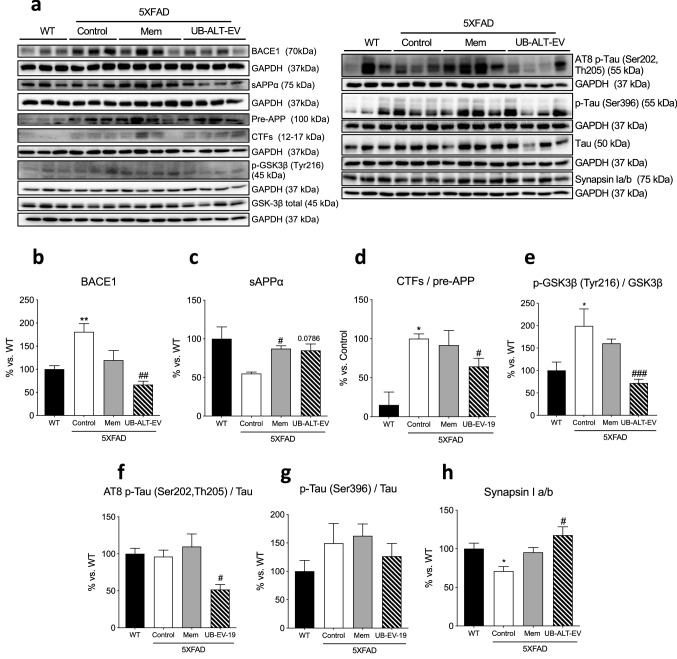
Representative western blot (**a**) and quantifications for BACE1 (**b**) sAPPα (**c**), ratio CTFs/pre-APP (**d**)**,** ratio p-GSK3β (Tyr216)/GSK3β (**e**), ratio AT8 p-Tau (Ser202, Th205)/Tau (**f**), ratio p-Tau/Tau (Ser396) (**g**)**,** and Synapsin I a/b (**h**). Values in bar graphs are adjusted to 100% for protein levels of the wild type (WT). Values are the mean ± Standard error of the mean (SEM); (n = 3 for WT and Control groups and *n* = 4 for Mem and UB-ALT-EV groups. For WT vs. 5XFAD Control groups, data were analyzed using a two-tailed Student’s *t* test, and for 5XFAD groups, a standard one-way ANOVA followed by Tukey post hoc analysis was performed. *p < 0.05; **p < 0.01 for WT vs. Control. ^#^*p* < 0.05; ^##^*p* < 0.01; ^###^*p* < 0.001 for Mem or UB-ALT-EV vs. Control

Since Aβ formation is a hallmark of AD, several APP protein processing proteins were studied. Significant higher protein levels of *β*-site amyloid precursor protein cleaving enzyme 1 (BACE1) were found in the 5XFAD Control group compared to the WT group. Strikingly, only UB-ALT-EV-treated mice were able to significantly reduce their protein levels compared to the 5XFAD Control group (Fig. 4a, b). Moreover, protein levels of soluble amyloid precursor protein α fragment (sAPPα), a non-amyloidogenic pathway marker, were significantly increased in both treated groups compared to the 5XFAD control group reaching similar levels of WT mice (Fig. 4a, c). In addition, when the c-terminal fragments (CTFs) were analyzed, the ratio CTFs/pre-APP results were significantly higher in the case of 5XFAD control group compared to the WT group. Likewise, a significant reduction of the ratio CTFs/pre-APP only was observed in UB-ALT-EV-treated animals compared to the 5XFAD control group (Fig. 4a, d). Those results suggest that after treatment with NMDAR antagonist, an amelioration of the amyloidogenic formation pathway, and fostering non-amyloidogenic pathway.

Since the hyper-phosphorylation of tau is a hallmark of AD, several protein levels related to tau pathology were evaluated. We mentioned above an increase in p25 (a coactivator of cyclin-dependent-kinase 5 (CDK5) tau kinase) and its diminution after UB-ALT-EV treatment (Fig. 3a, f). Pursuing tau kinases activity, we evaluated GSK3β quantifying the ratio p-GSK3β Tyr216/GSK3β and, remarkably, we only found a significant reduction in the UB-ALT-EV treated group in comparison with the 5XFAD Control group (Fig. 4a, e). Accordingly, only the UB-ALT-EV treatment significantly reduced protein levels of phosphorylated AT8 tau (Ser202, Th205) compared to the 5XFAD control group (Fig. 4a, f). Besides, p-Tau (Ser396) protein was reduced after UB-ALT-EV, but did not reach significance (Fig. 4a, g). All of the above results suggest that UB-ALT-EV prevented tau hyper-phosphorylation.

Synapsin I is a synaptic vesicle-associated protein downregulated in AD [40], and also, it has been associated with tau [41]. Indeed, synapsin I was found in significantly lower levels in the 5XFAD mice compared to the WT group (Fig. 4a, h). Interestingly, our results indicated that synapsin I protein levels were increased after treatment with UB-ALT-EV, but not with memantine (Fig. 4a, h).

### Reduced plaques deposition induced by NMDAR antagonists

**Fig. 5:**
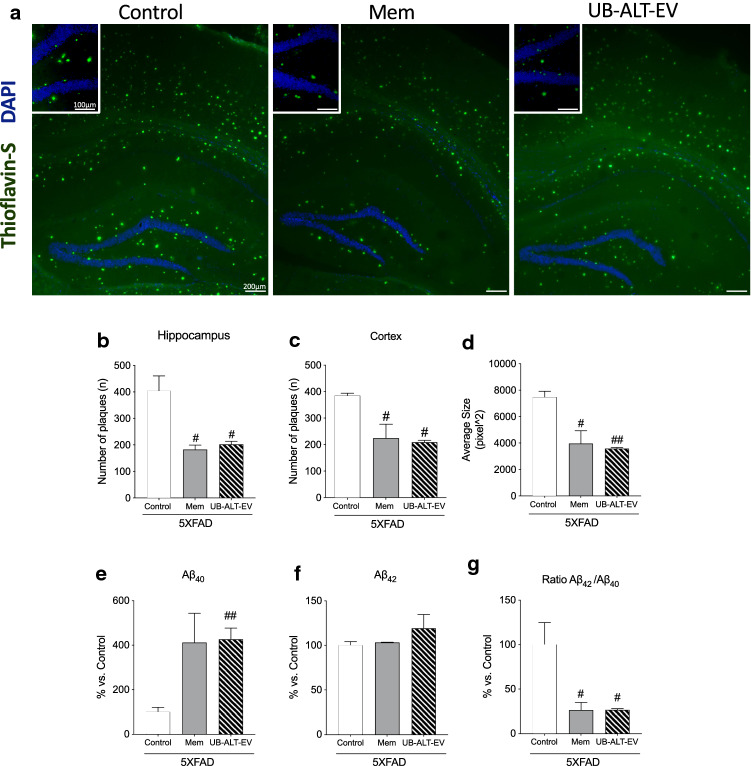
Thioflavin-S-positive staining (green) and DAPI (blue) in the 5XFAD hippocampus and cerebral cortex for Control, Mem and UB-ALT-EV(**a**). Quantification of Thioflavin-S positive staining in 5XFAD mice hippocampus (**b**) and cortex (**c**). Average Size of Thioflavin-S plaques measurement (**d**). Levels of amyloid-β_40_ levels (**e**). Levels of amyloid-β_42_ (**f**). Ratio of amyloid-β_42_/amyloid-β_40_ (**g**). Values are the mean ± Standard error of the mean (SEM); (n = 3 for each group). A standard one-way ANOVA followed by Tukey post hoc analysis was performed. ^#^*p* < 0.05; ^##^*p* < 0.01 for Mem or UB-ALT-EV vs. Control

Plaques were quantified by thioflavin-S staining (Fig. 5a) in 5XFAD mice hippocampus and cortex. Results highlighted the ability of the NMDAR antagonists’ treatments to reduce the amyloid burden compared to the 5XFAD control group (Fig. 5b, c). Remarkably, we found that treated animals with memantine or UB-ALT-EV presented smaller size plaques compared to the 5XFAD control group (Fig. 5d). Interestingly, our results showed a significant increase of Aβ_40_ levels in 5XFAD mice treated with NMDA receptor antagonist, whereas no changes occurred for Aβ_42_ (Fig. 5e, f). Therefore, Aβ_42_/Aβ_40_ ratios were significantly decreased after treatment compared with control group (Fig. 5g). These results highlighted the neuroprotective role of NMDAR antagonists in reducing an important hallmark of AD.

### NMDAR antagonist UB-ALT-EV treatment in 5XFAD mice induced changes in apoptosis and the autophagic process

**Fig. 6:**
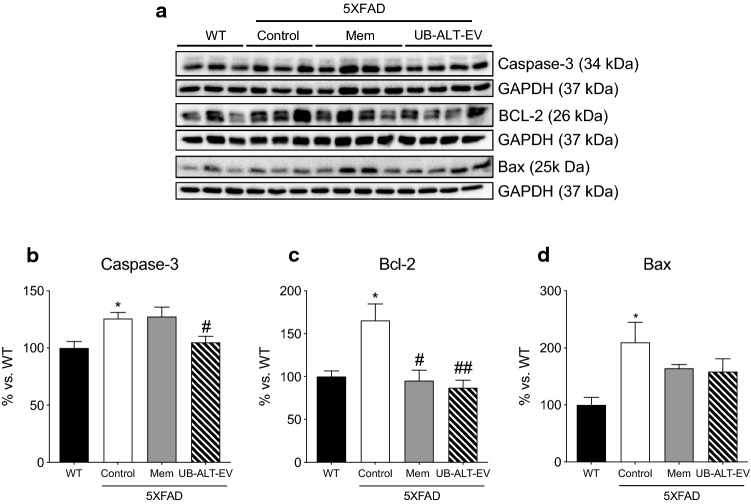
Representative western blot (**a**) and quantifications for Caspase-3 (**b**) Bcl-2 (**c**) and Bax (**d**). Values in bar graphs are adjusted to 100% for protein levels of the wild type (WT). Values are the mean ± Standard error of the mean (SEM); (*n* = 3 for WT and Control groups and n = 4 for Mem and UB-ALT-EV groups. For WT vs. 5XFAD Control groups, data were analyzed using a two-tail Student’s t test, and for 5XFAD groups, a standard one-way ANOVA followed by Tukey post hoc analysis was performed. *p < 0.05 for WT vs. Control. ^#^*p* < 0.05; ^##^*p* < 0.01 for Mem or UB-ALT-EV vs. Control

To further evaluate the neuroprotective effects of NMDAR antagonists in 5XFAD mice, the levels of apoptosis-related proteins were determined**.** Caspase-3 was evaluated as a critical apoptotic protein, being protein levels significantly higher in 5XFAD Control mice than in the WT group. UB-ALT-EV-treated 5XFAD mice showed a significant diminution in caspase-3 protein levels compared to the 5XFAD control group (Fig. 6a, b). By contrast, the SBDP 120 kDa fragment (a marker of caspase-3 activation) increased in a significant way in memantine-treated group compared to 5XFAD Control mice (Fig. 3a–d). Surprisingly, the anti-apoptotic protein B-cell lymphoma 2 (Bcl-2) increased in 5XFAD Control mice, but after NMDAR antagonists treatment, Bcl-2 protein levels reached WT mice levels (Fig. 6a, c). By contrast, the pro-apoptotic protein Bax was found increased in 5XFAD Control mice, and after NMDAR antagonist treatment was reestablished to the WT levels (Fig. 6a, d). These findings indicated decreased apoptotic process in 5XFAD by NMDAR antagonist treatment.

**Fig. 7:**
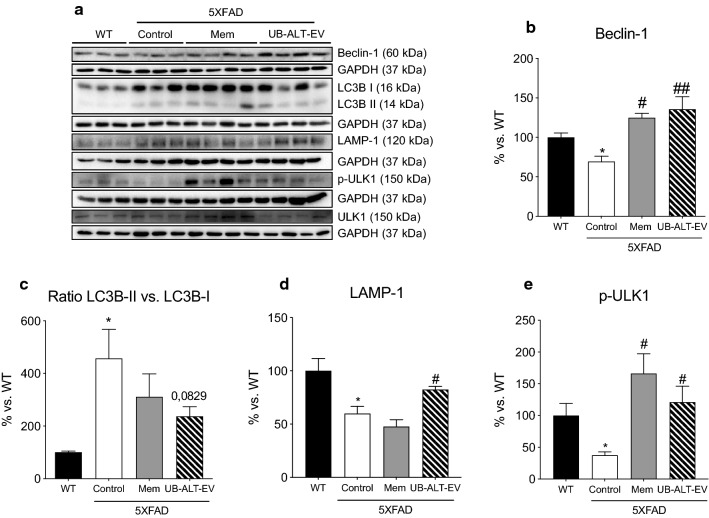
Representative western blot (**a**) and quantifications for Beclin-1 (**b**), ratio LC3B-II vs. LC3B-I (**c**), LAMP-1 (**d**), and ULK-1 (**e**). Values in bar graphs are adjusted to 100% for protein levels of the wild type (WT). Values are the mean ± Standard error of the mean (SEM) (*n* = 3 for WT and Control groups and *n* = 4 for Mem and UB-ALT-EV groups). For WT vs. 5XFAD Control groups, data were analyzed using a two-tailed Student’s t test, and for 5XFAD groups, a standard one-way ANOVA followed by Tukey post hoc analysis was performed. *p < 0.05 for WT vs. Control. ^#^*p* < 0.05; ^##^*p* < 0.01 for Mem or UB-ALT-EV vs. Control

Additionally, autophagic markers were evaluated. Unc-51-like kinase (ULK-1) and Beclin-1 are initiation markers of the autophagic process. A significant decrease in unc-51-like kinase and beclin-1 protein levels in 5XFAD compared with WT was demonstrated, therefore revealing a dysfunctional autophagy process. UB-ALT-EV and memantine treatments prevented those changes (Fig. 7a, b, e). However, p62, a protein necessary for phagophore generation, was unmodified throughout the experimental groups (data not shown). Then, microtubule-associated protein 1A/1B light chain 3 I and II (LC3B-I and LC3B-II), a well-known essential actor for autophagy, was evaluated. Usually, LC3B-II correlates with the number of autophagosomes. However, ratio LC3B-II/LC3B-I, was increased in transgenic mice and was partially diminished in UB-ALT-EV-treated mice (Fig. 7a, c). Lysosomal-associated membrane protein 1 (LAMP-1) was also studied as a marker for autophagolysosome formation. Our results showed a significant decrease in LAMP-1 protein levels in 5XFAD control mice in front of the WT mice, which were recovered to WT mice levels in 5XFAD mice treated with UB-ALT-EV. On the whole, results suggested that treatment increases autophagic flux in 5XFAD mice (Fig. 7a, d).

## Discussion

More effective pharmacological treatments or disease-modifying agents are urgently needed to slow down or prevent the progression of AD. Since memantine was approved in 2003 to treat patients with dementia and moderate-to-severe AD, no other drug has successfully passed clinical trials [42, 43]. Only very recently, the disease-modifying agent, aducanumab, has been conditionally approved by the FDA based on its presumed efficacy [44]. In any case, it is worth noting that, nowadays, symptomatic drugs, such as memantine, are still essential to treat the behavioral and cognitive alterations that appear during the disease.

Notwithstanding, growing evidence suggesting that targeting NMDARs could protect against excitotoxicity and may also alter other signaling cascades, promoting indirect neuroprotective effects that have not previously been studied. Therefore, the field may benefit from developing new NMDAR antagonists with an improved pharmacological profile and reduced side effects or neurotoxicity. Furthermore, the development of new antagonists has also been described in several in vitro and in vivo published reports carried out over the last decade in various AD mouse models, demonstrating the potential to target NMDARs [21, 37, 45, 46].

The present study was conducted to demonstrate the efficacy of UB-ALT-EV, a new BBB penetrant optimized NMDAR antagonist, to ameliorate cognitive impairment through the modulation of NMDAR-mediated neurodegenerative pathways promoted by Aβ pathology in the 5XFAD mouse model. In addition, we incorporate memantine as a gold standard to compare to the new compound, UB-ALT-EV. Of note, only two studies have been published describing the effects of memantine on 5XFAD mice, and to the best of our knowledge, none of them provides results about the effects of chronic low-dose memantine treatment on cognition, apoptosis, and autophagy in 5XFAD mice [23, 35].

First, we demonstrated the oral bioavailability for UB-ALT-EV and its ability to cross BBB when applied orally at a 5 mg/kg dose, confirming that it can reach the target in the brain in a sufficiently high concentration. As we expected, NMDAR antagonist’s treatment improved behavioral tasks, showing higher locomotor activity, and reducing alterations associated with anxiety-like behavior. In addition, we confirmed a firmly cognitive recovery for spatial memory deficits in 5XFAD mice treated with both NMDAR antagonists, confirming our results in working memory in 5XFAD mice model treated with UB-ALT-EV and memantine [47]. Thus, in line with those results, it has been described that NMDAR antagonist strategy displayed better cognitive performance, including behavioral abnormalities in AD mice [23–25, 35].

Regarding the mechanisms of action, it is assumed that NMDARs are pathologically overactivated in AD. Resulting in elevated intracellular levels of Ca^2+^, which activate a variety of downstream signaling pathways that promote neurodegeneration [48]. However, the presence of NMDAR2A subunit rather than NMDAR2B on NMDARs complex has been described to mediate neuroprotective pathways [49, 50]. We recently showed that NMDAR2A levels in 5XFAD mice were lower than WT, and UB-ALT-EV treatment was able to increase NMDAR2A subunit protein levels slightly but significantly [47]. Besides, we found that UB-ALT-EV, in a more effective way than memantine, increased tyrosine phosphorylation of NMDAR2B subunits. This event is known to prevent NMDARs’ internalization, and, consequently, fostering cell-survival, synaptic function, and cognitive improvement.

Another possible mechanism by which NMDAR antagonists could mediate neuroprotection is by reducing the calcium influx impacting the activity of calcium-dependent proteins [51]. In fact, NMDAR antagonists are known to modulate the calcium-mediated pathways related to neurodegenerative disorders like AD [52]. Then, we delve further into the modulation of calcium-dependent proteins by UB-ALT-EV and memantine in 5XFAD mice. Our experiments demonstrated that UB-ALT-EV significantly reduced calpain-1 levels and, importantly, its activation, as can be deducted by p25/p35 ratio decrease, pointing out a potent effect of this new NMDAR antagonist and suggesting a reduction of calcium signaling. The calpain-1 inhibition after UB-ALT-EV treatment was confirmed by the reduction of the *α*-spectrin SBDP 150 fragment protein levels in UB-ALT-EV-treated animals but not memantine-treated ones. This is consistent with a previous work that demonstrated a reduction in calpain activation after the treatment with NDMARs’ antagonists [53].

Moreover, we found that after NMDAR antagonist treatment, CaMKII, other calcium-dependent protein, phosphorylation levels decreased, supporting the decrease in calcium entry through NMDARs, and subsequently, CaMKII activation. Interestingly, it has been demonstrated that the decreased autophosphorylation of CaMKII recovered cognitive symptoms of dementia in mice by the use of memantine or donepezil [54]. Thus, the data support the hypothesis that calpain-1 and CaMKII activity modulation by UB-ALT-EV treatment might improve cell signaling and finally lead to reduced cognitive deficits presented by 5XFAD mice. Furthermore, we found increased levels of synapsin I, a synaptic vesicle-associated protein [55], after UB-ALT-EV treatment, that may explain partially the amelioration on 5XFAD mice cognitive status. Consistent with these findings, *SynI*^*−/−*^ mice showed spatial and emotional abnormalities through different behavioral and cognitive tests [56]. Furthermore, UB-ALT-EV treatment increased levels of postsynaptic density protein 95 (PSD95) in 5XFAD [47]. In whole, those findings suggested that UB-ALT-EV treatment mediates synapse improvement.

Of note, CaMKII activation coincides with pathological phosphorylation of tau in AD brains and is also activated by the disruption of calcium homeostasis [57] like it occurs in 5XFAD mice. As mentioned, tau pathology is associated with a complex modulatory network of proteins, in which CDK5 and GSK3β are the most prominent tau kinases [58]. Then, we evaluated the activity of GSK3β through the ratio of p-GSK3β Tyr216/total GSK3β as well as tau phosphorylation levels. Our results showed that UB-ALT-EV, but not memantine, caused a diminution in GSK3β activity and AT8 levels in 5XFAD. These results reflect the modulation of this AD mark by UB-ALT-EV, linking GSK3β activity to NMDARs functionality which differs from that of classical NMDAR antagonists [25]. Moreover, we found that both treatments, UB-ALT-EV and memantine, ameliorated Aβ pathology in treated 5XFAD mice, as demonstrated by a reduction in the number and size of plaques, and also by the reduction of the Aβ_42_/Aβ_40_ ratio [59]. Likewise, we found about 4 × increased levels of Aβ_40_ in both treated groups. Of note, it is well-established that this amyloid fragment inhibits Aβ deposition in vivo [60], suggesting in part the mechanism by which Aβ deposition might be reduced after both treatments. Furthermore, the improvement of amyloidogenic pathology after treatment with UB-ALT-EV was accompanied by beneficial effects on APP processing as evidenced by protein levels for BACE1, sAPPα, and the CTFs/pre-APP ratio. Those results support the hypothesis that disease progression in 5XFAD mice is modified by NMDAR antagonists’ treatment.

The calpain protease system and autophagy are strongly interconnected, and modifications in calpain expression induce changes in autophagic processes. Concretely, it is known that calpains can cleave proteins involved in autophagy [61]. In 5XFAD, autophagy was impaired by altered levels of ULK-1, beclin-1, LC3B, and LAMP-1 proteins, indicating that the autophagic process was unfinished. Interestingly, calpain-1 is implicated in the proteolytic cleavage of beclin-1, impeding the initiation of autophagosome formation [62]; in fact, a decrease in beclin-1 levels has been described in AD [63]. Interestingly, it has been demonstrated that the overexpression of beclin-1 enhances autophagic process initiation, reducing Aβ levels and improving cognition in different animal models, including 5XFAD mice [63, 64]. In this line of evidence, treatment of 5XFAD mice with UB-ALT-EV increased beclin-1 protein levels, probably due to the decreased calpain-1 proteolytic activity. Both phenomena allowed the initiation of the beclin-1-mediated autophagosome formation, promoting the initiation of the autophagic process jointly with the increased protein levels of ULK-1, which plays a critical role during the early stages of autophagy [65]. In the next step of autophagy, 5XFAD mice revealed an accumulation of LC3B-II, a promoter of phagophore formation [66] and low levels of LAMP-1, that plays an important role in lysosome biogenesis [67], indicating a defect in the autophagosome degradation. Interestingly, the reduced levels of LC3B-II vs. LC3B-I ratio observed in the 5XFAD-treated groups could be identified as an amelioration of the autophagosome accumulation. Moreover, UB-ALT-EV treatment increased LAMP-1 protein levels, enhancing the autophagolysosome generation and allowing the completion of effective and proper autophagy process that would improve cell function by removing cellular debris such as aberrant protein accumulation. Likewise, these findings are in accordance with the reduced plaques’ deposition results. Overall, it was reported that autophagy enhancement can drive a reduction in Aβ levels and apoptosis, thereby inhibiting apoptosis [17–19, 68]. Our results suggested that UB-ALT-EV would regulate apoptosis and autophagic process supporting its neuroprotective effect through NMDAR antagonism beyond memantine.

Remarkably, the cross-talk between calpain-caspase-3 apoptotic signaling pathways has been related to AD through NMDARs’ overactivation [69]. Caspase-3 protein levels were slightly modified in 5XFAD mice and decreased under UB-ALT-EV treatment, but no changes in SBDP 120 kDa fragments were determined; however, it must be kept in mind that activation of caspase-3 is not directly associated with calcium increases. Furthermore, it was demonstrated that apoptosis could be promoted by autophagy in AD patients [70, 71], whereby the reduced levels of caspase-3 found here could be explained by the increased autophagic process induced by UB-ALT-EV treatment. Supporting those results, it was found that memantine could attenuate cell apoptosis by inhibiting the calpain-caspase-3 after ischemic stroke intervention [15].

Regarding the Bcl-2, increased levels are well described in AD brains [72] and in amyloid containing brain regions of mice overexpressing APP[73]. This increase in an anti-apoptotic protein has been proposed as a response to neuronal insults, such as oxidative stress and the presence of Aβ [73]. Previous reports demonstrated high levels of Bcl-2 in mice models of AD that were reduced after treatment with neuroprotective drugs [28, 74]. Accordingly, Bcl-2 expression after treatment confirmed the influence of NMDAR antagonists on regulating the apoptotic process.

## Conclusions

**Fig. 8:**
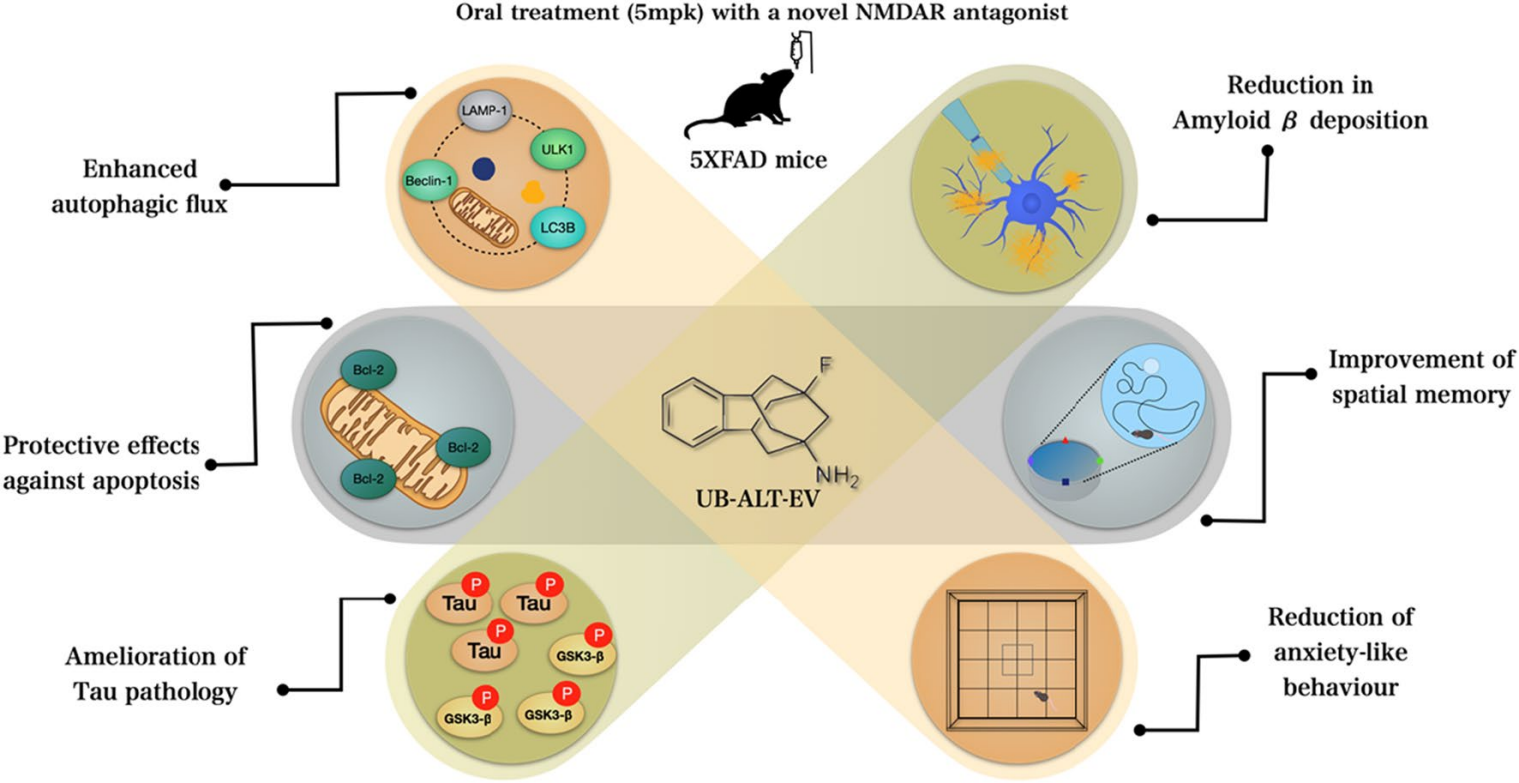
Illustrative cartoon of effects of UB-ALT-EV in 6-months-old 5XFAD mice

Based on our results, it can be concluded that chronic treatment with UB-ALT-EV resulted in neuroprotective effects on 5XFAD mice, ameliorating the tau and amyloid pathology, as well as cognitive improvements (Fig. 8). Together with those recently reported, these findings propose new NMDAR antagonist drug research opportunities on more effective treatments for fighting against cognitive impairment and AD.

## Conflict of interest

The authors have no relevant financial or non-financial interests to disclose. *Financial interests***: **Authors declare that they have no financial interests.

## Ethical approval

This study was performed in line with the principles of the Declaration of Helsinki, according to European Community Council Directive 86/609/EEC, and was approved by the Institutional Animal Care and Use Committee of the University of Barcelona (670/14/8102, approved at 14 November 2014) and by Generalitat de Catalunya, Spain (10291, approved at 28 January 2018).

## Consent for publication

Not applicable.

## Supplementary Information

Below is the link to the electronic supplementary material.

**Fig. S1 **Body weight measurement over the last six weeks of the study for WT and 5XFAD mice. (PDF 36 KB)

Supplementary file2. (DOCX 15 KB)

## Data Availability

The datasets generated during and/or analyzed during the current study are not publicly available due to are standard raw data for RT-PCR, Western blot, and IHC image quantification, etc., but are available from the corresponding author on reasonable request.

## References

[1] Parsons CG, Danysz W, Quack G (1999). Memantine is a clinically well tolerated *N*-methyl-d-aspartate (NMDA) receptor antagonist—a review of preclinical data. Neuropharmacol.

[2] Zhang Y, Li P, Feng J, Wu M (2016). Dysfunction of NMDA receptors in Alzheimer’s disease. Neurol Sci.

[3] Filali M, Lalonde R, Rivest S (2011). Subchronic memantine administration on spatial learning, exploratory activity, and nest-building in an APP/PS1 mouse model of Alzheimer’s disease. Neuropharmacology.

[4] Kumar A (2015). NMDA receptor function during senescence: Implication on cognitive performance. Front Neurosci.

[5] Wittenauer BR, Smith L (2013) Priority medicines for Europe and the World “A Public Health Approach to Innovation” Update on 2004 Background Paper. B6.12; World Health Organization

[6] Mattson MP (2004). Pathways towards and away from Alzheimer’s disease. Nature.

[7] World Health Organization (2016). WHO | Dementia: a public health priority. World Heal. Organ.

[8] Viola KL, Klein WL (2015). Amyloid β oligomers in Alzheimer’s disease pathogenesis, treatment, and diagnosis. Acta Neuropathol.

[9] Dickson DW (1997). Neuropathological diagnosis of Alzheimer’s disease: a perspective from longitudinal clinicopathological studies. Neurobiol Aging.

[10] L’Episcopo F, Drouin-Ouellet J, Tirolo C, Pulvirenti A, Giugno R, Testa N (2016). GSK-3β-induced Tau pathology drives hippocampal neuronal cell death in Huntington’s disease: involvement of astrocyte–neuron interactions. Cell Death Dis.

[11] Lv X, Li Q, Mao S, Qin L, Dong P (2020). The protective effects of memantine against inflammation and impairment of endothelial tube formation induced by oxygen-glucose deprivation/reperfusion. Aging (Albany NY).

[12] Song G, Li Y, Lin L, Cao Y (2015). Anti-autophagic and anti-apoptotic effects of memantine in a SH-SY5Y cell model of Alzheimer’s disease via mammalian target of rapamycin-dependent and -independent pathways. Mol Med Rep.

[13] Pietá Dias C, Martinsde Lima MN, Presti-Torres J, Dornelles A, Garcia VA, Siciliani Scalco F (2007). Memantine reduces oxidative damage and enhances long-term recognition memory in aged rats. Neuroscience.

[14] Ndountse LT, Chan HM (2009). Role of *N*-methyl-d-aspartate receptors in polychlorinated biphenyl mediated neurotoxicity. Toxicol Lett.

[15] Chen B, Wang G, Li W, Liu W, Lin R, Tao J (2017). Memantine attenuates cell apoptosis by suppressing the calpain-caspase-3 pathway in an experimental model of ischemic stroke. Exp Cell Res Exp Cell Res.

[16] Shen H, Zhu H, Panja D, Gu Q, Li Z (2020). Autophagy controls the induction and developmental decline of NMDAR-LTD through endocytic recycling. Nat Commun.

[17] Spilman P, Podlutskaya N, Hart MJ, Debnath J, Gorostiza O, Bredesen D (2010). Inhibition of mTOR by rapamycin abolishes cognitive deficits and reduces amyloid-β levels in a mouse model of Alzheimer’s disease. PLoS ONE.

[18] Tan CC, Yu JT, Tan MS, Jiang T, Zhu XC, Tan L (2014). Autophagy in aging and neurodegenerative diseases: implications for pathogenesis and therapy. Neurobiol Aging.

[19] Zhu XC, Yu JT, Jiang T, Tan L (2013). Autophagy modulation for Alzheimer’s disease therapy. Mol Neurobiol.

[20] Kishi T, Matsunaga S, Oya K, Nomura I, Ikuta T, Iwata N (2017). Memantine for Alzheimer’s disease: an updated systematic review and meta-analysis. J Alzheimer’s Dis.

[21] Liu MY, Wang S, Yao WF, Zhang ZJZ, Zhong X, Sha L (2014). Memantine improves spatial learning and memory impairments by regulating NGF signaling in APP/PS1 transgenic mice. Neuroscience.

[22] Wang X, Blanchard J, Grundke-Iqbal I, Iqbal K (2015). Memantine attenuates Alzheimer’s disease-like pathology and cognitive impairment. PLoS ONE.

[23] Jürgenson M, Zharkovskaja T, Noortoots A, Morozova M, Beniashvili A, Zapolski M (2019). Effects of the drug combination memantine and melatonin on impaired memory and brain neuronal deficits in an amyloid-predominant mouse model of Alzheimer’s disease. J Pharm Pharmacol.

[24] Scholtzova H, Wadghiri YZ, Douadi M, Sigurdsson EM, Li Y-S, Quartermain D (2008). Memantine Leads to Behavioral Improvement and Amyloid Reduction in Alzheimer’s-Disease-Model Transgenic Mice Shown as by Micromagnetic Resonance Imaging. J Neurosci Res.

[25] Martinez-Coria H, Green KN, Billings LM, Kitazawa M, Albrecht M, Rammes G (2010). Memantine improves cognition and reduces Alzheimer’s-like neuropathology in transgenic mice. Am J Pathol.

[26] Briggs R, Kennelly SP, O’Neill D (2016). Drug treatments in Alzheimer’s disease. Clin Med J R Coll Physicians London.

[27] Mufson EJ, Counts SE, Perez SE, Ginsberg SD (2008). Cholinergic system during the progression of Alzheimer’s disease: therapeutic implications. Expert Rev Neurother.

[28] Companys-Alemany J, Turcu AL, Bellver-Sanchis A, Loza MI, Brea JM, Canudas AM (2020). A novel NMDA receptor antagonist protects against cognitive decline presented by senescent mice. Pharmaceutics.

[29] Leiva R, Phillips MB, Turcu AL, Gratacòs-Batlle E, León-García L, Sureda FX (2018). Pharmacological and electrophysiological characterization of novel NMDA receptor antagonists. ACS Chem Neurosci.

[30] Valverde E, Sureda FX, Vázquez S (2014). Novel benzopolycyclic amines with NMDA receptor antagonist activity. Bioorg Med Chem.

[31] Girard SD, Jacquet M, Baranger K, Migliorati M, Escoffier G, Bernard A (2014). Onset of hippocampus-dependent memory impairments in 5XFAD transgenic mouse model of Alzheimer’s disease. Hippocampus.

[32] Griñán-Ferré C, Sarroca S, Ivanova A, Puigoriol-Illamola D, Aguado F, Camins A (2016). Epigenetic mechanisms underlying cognitive impairment and Alzheimer disease hallmarks in 5XFAD mice. Aging (Albany NY).

[33] Lazic D, Tesic V, Jovanovic M, Brkic M, Milanovic D, Zlokovic BV (2020). Every-other-day feeding exacerbates inflammation and neuronal deficits in 5XFAD mouse model of Alzheimer’s disease. Neurobiol Dis.

[34] Li N, Li Y, Li L-J, Zhu K, Zheng Y, Wang X-M (2019). Glutamate receptor delocalization in postsynaptic membrane and reduced hippocampal synaptic plasticity in the early stage of Alzheimer’s disease. Neural Regen Res.

[35] Devi L, Ohno M (2016). Cognitive benefits of memantine in Alzheimer’s 5XFAD model mice decline during advanced disease stages. Pharmacol Biochem Behav.

[36] Vina J, Lloret A (2010). Why women have more Alzheimer’s disease than men: gender and mitochondrial toxicity of amyloid-beta peptide. J Alzheimers Dis.

[37] Zhou X, Wang L, Xiao W, Su Z, Zheng C, Zhang Z (2019). Memantine improves cognitive function and alters hippocampal and cortical proteome in triple transgenic mouse model of Alzheimer’s disease. Exp Neurobiol.

[38] Seibenhener ML, Wooten MC (2015). Use of the open field maze to measure locomotor and anxiety-like behavior in mice. J Vis Exp.

[39] Nunez J (2008). Morris water maze experiment.

[40] Marsh J, Bagol SH, Williams RSB, Dickson G, Alifragis P (2017). Synapsin I phosphorylation is dysregulated by beta-amyloid oligomers and restored by valproic acid. Neurobiol Dis US.

[41] Robbins M, Clayton E, Kaminski Schierle GS (2021). Synaptic tau: a pathological or physiological phenomenon?. Acta Neuropathol Commun.

[42] Muir KW (2006). Glutamate-based therapeutic approaches: clinical trials with NMDA antagonists. Curr Opin Pharmacol.

[43] Ikonomidou C, Turski L (2002). Why did NMDA receptor antagonists fail clinical trials for stroke and traumatic brain injury?. Lancet Neurol.

[44] Mahase E (2021). FDA approves controversial Alzheimer’s drug despite uncertainty over effectiveness. BMJ.

[45] Nagakura A, Shitaka Y, Yarimizu J, Matsuoka N (2013). Characterization of cognitive deficits in a transgenic mouse model of Alzheimer’s disease and effects of donepezil and memantine. Eur J Pharmacol.

[46] Sun D, Chen J, Bao X, Cai Y, Zhao J, Huang J (2015). Protection of radial glial-like cells in the hippocampus of APP/PS1 Mice: a novel mechanism of memantine in the treatment of Alzheimer’s disease. Mol Neurobiol.

[47] Turcu AL, Companys-Alemany J, Phillips MB, Patel DS, Griñán-Ferré C, Loza MI (2022). Design, synthesis, and in vitro and in vivo characterization of new memantine analogs for Alzheimer’s disease. Eur J Med Chem.

[48] Liu J, Chang L, Song Y, Li H, Wu Y (2019). The role of NMDA receptors in Alzheimer’s disease. Front Neurosci.

[49] Proctor DT, Coulson EJ, Dodd PR (2011). Post-synaptic scaffolding protein interactions with glutamate receptors in synaptic dysfunction and Alzheimer’s disease. Prog Neurobiol.

[50] Chen M, Lu TJ, Chen XJ, Zhou Y, Chen Q, Feng XY (2008). Differential roles of NMDA receptor subtypes in ischemic neuronal cell death and ischemic tolerance. Stroke.

[51] Mahaman YAR, Huang F, Afewerky HK, Maibouge TMS, Ghose B, Wang X (2019). Involvement of calpain in the neuropathogenesis of Alzheimer’s disease. Med Res Rev.

[52] Wang R, Reddy PH (2017). Role of Glutamate and NMDA receptors in Alzheimer’s disease. J Alzheimer’s Dis.

[53] Tanqueiro SR, Ramalho RM, Rodrigues TM, Lopes LV, Sebastião AM, Diógenes MJ (2018). Inhibition of NMDA receptors prevents the loss of BDNF function induced by amyloid β. Front Pharmacol.

[54] Yabuki Y, Matsuo K, Hirano K, Shinoda Y, Moriguchi S, Fukunaga K (2017). Combined memantine and donepezil treatment improves behavioral and psychological symptoms of dementia-like behaviors in olfactory bulbectomized mice. Pharmacology.

[55] Sudhof T, Czernik A, Kao H, Takei K (1989). Synapsins: mosaics of shared and individual domains in a family of synaptic vesicle phosphoproteins. Science (80–).

[56] Corradi A, Zanardi A, Giacomini C, Onofri F, Valtorta F, Zoli M (2008). Synapsin-I- and synapsin-II-null mice display an increased age-dependent cognitive impairment. J Cell Sci.

[57] Oka M, Fujisaki N, Maruko-Otake A, Ohtake Y, Shimizu S, Saito T (2017). Ca2+/calmodulin-dependent protein kinase II promotes neurodegeneration caused by tau phosphorylated at Ser262/356 in a transgenic Drosophila model of tauopathy. J Biochem.

[58] Lauretti E, Dincer O, Praticò D (2020). Glycogen synthase kinase-3 signaling in Alzheimer’s disease. Biochim Biophys acta Mol Cell Res.

[59] Oakley H, Cole SL, Logan S, Maus E, Shao P, Craft J (2006). Intraneuronal β-amyloid aggregates, neurodegeneration, and neuron loss in transgenic mice with five familial Alzheimer’s disease mutations: potential factors in amyloid plaque formation. J Neurosci.

[60] Kim J, Onstead L, Randle S, Price R, Smithson L, Zwizinski C (2007). Aβ40 inhibits amyloid deposition in vivo. J Neurosci.

[61] Weber JJ, Pereira Sena P, Singer E, Nguyen HP (2019). Killing two angry birds with one stone: autophagy activation by inhibiting calpains in neurodegenerative diseases and beyond. Biomed Res Int.

[62] Russo R, Berliocchi L, Adornetto A, Varano G, Cavaliere F, Nucci C (2011). Calpain-mediated cleavage of Beclin-1 and autophagy deregulation following retinal ischemic injury in vivo. Cell Death Dis.

[63] Pickford F, Masliah E, Britschgi M, Lucin K, Narasimhan R, Jaeger PA (2008). The autophagy-related protein Beclin 1 shows reduced expression in early Alzheimer disease and regulates amyloid beta accumulation in mice. J Clin Invest.

[64] Rocchi A, Yamamoto S, Ting T, Fan Y, Sadleir K, Wang Y (2017). A Becn1 mutation mediates hyperactive autophagic sequestration of amyloid oligomers and improved cognition in Alzheimer’s disease. PLoS Genet.

[65] Zachari M, Ganley IG (2017). The mammalian ULK1 complex and autophagy initiation. Essays Biochem.

[66] Lee Y-K, Lee J-A (2016). Role of the mammalian ATG8/LC3 family in autophagy: differential and compensatory roles in the spatiotemporal regulation of autophagy. BMB Rep.

[67] Cheng X-T, Xie Y-X, Zhou B, Huang N, Farfel-Becker T, Sheng Z-H (2018). Revisiting LAMP1 as a marker for degradative autophagy-lysosomal organelles in the nervous system. Autophagy.

[68] Caccamo A, Majumder S, Richardson A, Strong R, Oddo S (2010). Molecular interplay between mammalian target of rapamycin (mTOR), amyloid-β, and Tau: effects on cognitive impairments. J Biol Chem.

[69] Carvajal FJ, Mattison HA, Cerpa W (2016). Role of NMDA receptor-mediated glutamatergic signaling in chronic and acute neuropathologies. Kang KD, editor. Neural Plast.

[70] Louneva N, Cohen JW, Han L-YY, Talbot K, Wilson RS, Bennett DA (2008). Caspase-3 is enriched in postsynaptic densities and increased in Alzheimer’s disease. Am J Pathol.

[71] Nixon RA, Wegiel J, Kumar A, Yu WH, Peterhoff C, Cataldo A (2005). Extensive involvement of autophagy in Alzheimer disease: an immuno-electron microscopy study. J Neuropathol Exp Neurol.

[72] Callens M, Kraskovskaya N, Derevtsova K, Annaert W, Bultynck G, Bezprozvanny I (2021). The role of Bcl-2 proteins in modulating neuronal Ca2+ signaling in health and in Alzheimer’s disease. Biochim Biophys Acta - Mol Cell Res.

[73] Karlnoski R, Wilcock D, Dickey C, Ronan V, Gordon MN, Zhang W (2007). Up-regulation of Bcl-2 in APP transgenic mice is associated with neuroprotection. Neurobiol Dis.

[74] Vasilopoulou F, Griñán-Ferré C, Rodríguez-Arévalo S, Bagán A, Abás S, Escolano C (2021). I2 imidazoline receptor modulation protects aged SAMP8 mice against cognitive decline by suppressing the calcineurin pathway. GeroScience.

