# Supplementary figures and images for: NMDA receptor antagonists reduce amyloid-β deposition by modulating calpain-1 signaling and autophagy, rescuing cognitive impairment in 5XFAD mice
Source: Cell Mol Life Sci. 2022 Jul 9;79(8):408. doi: 10.1007/s00018-022-04438-4 (PMC9271115; doi:10.1007/s00018-022-04438-4)

Figure S1.

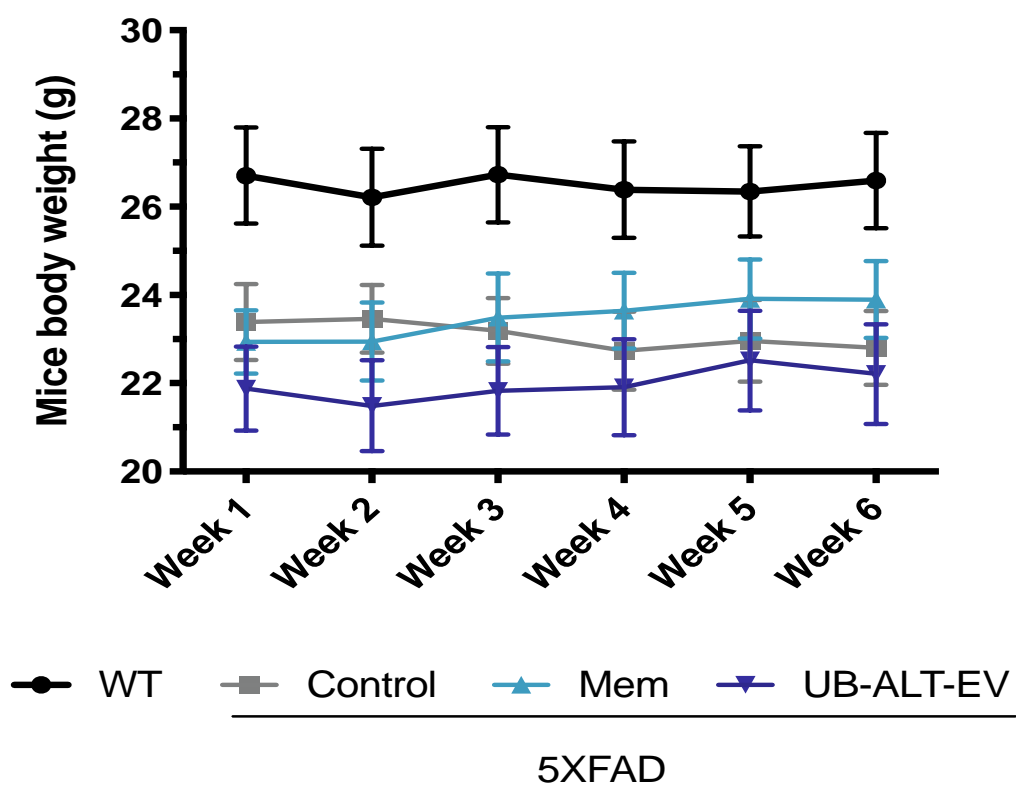

Supplement: Supplementary file 1 — Fig. S1 Body weight measurement over the last six weeks of the study for WT and 5XFAD mice. (PDF 36 KB) [file 18_2022_4438_MOESM1_ESM.pdf]
