# Supplementary material for: NMDA receptor antagonists reduce amyloid-β deposition by modulating calpain-1 signaling and autophagy, rescuing cognitive impairment in 5XFAD mice
Source: Cell Mol Life Sci. 2022 Jul 9;79(8):408. doi: 10.1007/s00018-022-04438-4 (PMC9271115; doi:10.1007/s00018-022-04438-4)
Supplement: Supplementary file 2 — Supplementary file2. (DOCX 15 KB) [file 18_2022_4438_MOESM2_ESM.docx]

Table S1: Antibodies used for Western blotting experiments

| Antibodies | Host | Source | | | Dilution |
| --- | --- | --- | --- | --- | --- |
| α-Spectrin alpha chain | Mouse | | Millipore/MAB1622 | 1:1000 | |
| APP C terminal fragment | Mouse | | Covance / SIG-39152 | 1:1000 | |
| BACE | Rabbit | | Cell Signaling / #5606 | 1:1000 | |
| Bax | Rabbit | | Cell Signaling / #2772 | 1:1000 | |
| BCL-2 | Rabbit | | Cell Signaling / #2870 | 1:1000 | |
| Beclin-1 | Rabbit | | Cell Signaling / #3495 | 1:1000 | |
| Calpain-1 | Rabbit | | Bio-Rad / AHP2443 | 1:1000 | |
| CaMKII | Rabbit | | Abcam / ab52476 | 1:1000 | |
| CaMKII (Phospho-Thr286) | Rabbit | | Signalway Antibody / #11287 | 1:1000 | |
| Caspase-3 | Rabbit | | BD Transduction Laboratories / C31720 | 1:1000 | |
| GAPDH | Mouse | | Millipore / #MAB374 | 1:5000 | |
| GSK3β | Rabbit | | Cell Signaling / #9315 | 1:1000 | |
| GSK3β (Phospho-Y216) | Rabbit | | Abcam / ab75745 | 1:1000 | |
| LAMP1 | Mouse | | Santa Cruz Biotechnology / sc-19992 | 1:500 | |
| LC3B | Rabbit | | Cell Signaling / #2775 | 1:1000 | |
| p25/35 | Rabbit | | Cell Signaling / #2680 | 1:1000 | |
| Tau Total | Rabbit | | GeneTex/GTX112981 | 1:1000 | |
| Tau (phospho-Ser396) | Rabbit | | Invitrogen/#44-752G | 1:1000 | |
| Tau (phosphor-Ser202, Thr205)(AT8) | Mouse | | Invitrogen/ #MN1020 | 1:1000 | |
| sAPPα | Rabbit | | Covance / SIG-39139-005 | 1:1000 | |
| Synapsin Ia | Mouse | | Santa Cruz Biotechnology / sc-136086 | 1:500 | |
| ULK1 | Mouse | | Santa Cruz Biotechnology / sc-390904 | 1:500 | |
| ULK1 (Phospho-Ser757) | Rabbit | | Cell Signaling / #6888 | 1:1000 | |
| anti-mouse HRP conjugated | Goat | | Bio-Rad / 170-5047 | 1:2000 | |
| anti-rabbit HRP conjugated | Goat | | Bio-Rad / 170-6515 | 1:2000 | |
